# ACE2 Nascence, trafficking, and SARS-CoV-2 pathogenesis: the saga continues

**DOI:** 10.1186/s40246-021-00304-9

**Published:** 2021-01-29

**Authors:** Sally Badawi, Bassam R. Ali

**Affiliations:** 1 Department of Genetics and Genomics, College of Medicine and Health Sciences, United Arab Emirates University, Al-Ain, United Arab Emirates; 2 Zayed Centre for Health sciences, United Arab Emirates University, Al-Ain, United Arab Emirates

**Keywords:** Angiotensin-converting enzyme 2 (ACE2), Trafficking, Localization, SARS-CoV-2, COVID-19

## Abstract

With the emergence of the novel coronavirus SARS-CoV-2 since December 2019, more than 65 million cases have been reported worldwide. This virus has shown high infectivity and severe symptoms in some cases, leading to over 1.5 million deaths globally. Despite the collaborative and concerted research efforts that have been made, no effective medication for COVID-19 (coronavirus disease-2019) is currently available. SARS-CoV-2 uses the angiotensin-converting enzyme 2 (ACE2) as an initial mediator for viral attachment and host cell invasion. ACE2 is widely distributed in the human tissues including the cell surface of lung cells which represent the primary site of the infection. Inhibiting or reducing cell surface availability of ACE2 represents a promising therapy for tackling COVID-19. In this context, most ACE2–based therapeutic strategies have aimed to tackle the virus through the use of angiotensin-converting enzyme (ACE) inhibitors or neutralizing the virus by exogenous administration of ACE2, which does not directly aim to reduce its membrane availability. However, through this review, we present a different perspective focusing on the subcellular localization and trafficking of ACE2. Membrane targeting of ACE2, and shedding and cellular trafficking pathways including the internalization are not well elucidated in literature. Therefore, we hereby present an overview of the fate of newly synthesized ACE2, its post translational modifications, and what is known of its trafficking pathways. In addition, we highlight the possibility that some of the identified ACE2 missense variants might affect its trafficking efficiency and localization and hence may explain some of the observed variable severity of SARS-CoV-2 infections. Moreover, an extensive understanding of these processes is necessarily required to evaluate the potential use of ACE2 as a credible therapeutic target.

## Introduction

Following the discovery of renin- and angiotensin-converting enzyme (ACE) [1, 2], the understanding of the renin-angiotensin system (RAS) has been greatly improved by the uncovering of associated receptors, enzymes, and protein complexes. Twenty years ago, in an approach of searching for human ACE homolog, two independent groups have identified angiotensin-converting enzyme 2 (ACE2) that shares a common ancestor with ACE, with 42% sequence identity [3, 4]. ACE2 expression disruption studies have identified ACE2 as an important regulator of the blood pressure and cardiovascular functions through its role in the renin-angiotensin system that counteracts ACE functions [5].

With the emergence of the severe acute respiratory syndrome (SARS), significant attention has been given to ACE2 due to its involvement as the host’s cellular receptor that mediates the initiation of the viral infection. Consequently, since 2002, approximately 4000 ACE2–related articles have been published where the majority corresponds to 2020 as revealed by a PubMed database search. In 2019, a novel coronavirus has emerged, known as SARS-CoV-2, causing the new coronavirus disease 2019 (termed COVID-19) and leading to unprecedented economic and health burden world-wide. Similar to its predecessor SARS-CoV and unlike MERS-CoV, the spike S protein of SARS-CoV-2 mediates the viral attachment and entry into the host cell by binding to its target primary receptor, the ACE2 [6, 7]. To facilitate viral fusion and entry, SARS-CoV-2 spike protein is proteolytically cleaved via cellular proteases into two subunits, S1 responsible for viral binding through its receptor-binding domain and S2 facilitating viral fusion and entry to the cell membrane [6]. Moreover, aside from ACE2, other alternative receptors/co-receptors and cellular proteases were also implicated in SARS-CoV-2 pathogenesis. Among these identified receptors are, CD147, known as the basic immunoglobulin or Basigin [8, 9], neuropilin-1 (NRP1) [10], the MERS-CoV receptor dipeptidyl peptidase 4 (DPP-4) [11, 12], angiotensin II receptor type 2 (AGTR2) [13], alanyl aminopeptidase (ANPEP) [14], and glutamyl aminopeptidase (ENPEP) [14]. Furthermore, the classical viral entry through the ACE2 receptor remains the extensively studied model. Consequently, the accumulated acquired knowledge of ACE2 has led to several therapeutic interventions to date such as the introduction of recombinant ACE2 protein as a hypertensive therapeutic target in 2009 [15] and a potent therapeutic target for SARS-related viruses [16–18]. However, some discrepancies and unknowns still exist, and further investigations are therefore required. In this context, throughout this review, we will present and discuss what is currently known about ACE2 biogenesis, regulation, and polymorphism with the emphasis on the gaps in our understanding of its intracellular trafficking and the potential of its use as a therapeutic target for COVID-19.

### Evolutionary history of ACE2 and its relation to coronaviruses

Controlling and preventing the infectious diseases in humans highly demand understanding and tracking their zoonotic origin. Earlier studies have suggested that coronavirus family, including both SARS-CoV-1 and MERS-CoV, are able to infect different species [19]. Coronaviruses were shown to infect humans either directly or indirectly through intermediate species. Phylogenic profiling has shown that bats represent the likely natural reservoir of SARS-CoV-1 and MERS-CoV [20, 21]. Of interest, intermediate species like the palm civets and camels were identified to be major transmission sources from animals to humans [22, 23]. Molecular evolution studies and phylogenic analysis of SARS-CoV-2 and ACE2 strongly suggest that SARS-CoV-2 might have crossed the species-specific barrier, with pangolins representing an intermediate species and bat as the main reservoir [24, 25]. ACE2 profiling and pattern conservation analysis have displayed that it is highly conserved within vertebrates, noting that this conservation shows high divergence as the evolutionary distance increases from Homo sapiens [26]. In addition, molecular characterization and sequence alignment analysis of ACE2 show that human and non-human primates display a low evolution rate with high similarity across species except for chicken [27]. Different studies suggested that this observed pattern of human ACE2 utilization might be explained by ancestral recombination events of viruses leading to different strains of the virus and enabling its transmission to humans and across species [28, 29]. Although MERS and SARS coronaviruses infect cells via different receptors, studies display a regulatory and interactive relationship between ACE2 and the MERS-CoV receptor, dipeptidyl peptidase-4 (DPP-4) [12, 30]. Similarly, evolutionary studies of DPP-4 show that it has undergone an adaptive evolution that has consequently allowed the transmission of MERS-CoV across species [31]. In addition to its receptor role, DPP-4 represents a T lymphocyte surface marker [32] that is involved in activating the immune response during infections. A combination therapy inhibiting DPP-4 and reducing ACE2 expression was hypothesized to combat SARS-CoV-2 infection via regulating the associated inflammatory reaction [33]. Interestingly, in a single cell RNA-seq analysis of 13 human tissues, DPP-4 has displayed a highly significant co-expression pattern with ACE2 [14]. Additionally, and like ACE2, DPP-4 can either be membrane-bound or shed into a soluble form. Similar to what happens during MERS-CoV infections, soluble circulating level of DPP-4 (sDPP-4) was detected at a reduced level in hospitalized patients with severe COVID-19 symptoms [30]. It is worth noting that Amati et al. have recently shown that in addition to ACE2, DPP-4 might represent a genomic biomarker that displays an increased expression profile in nasopharyngeal and oropharyngeal swabs [34]. Altogether, these data provide evidence about the possibility of DPP-4 to act as a co-receptor for SARS-CoV-2; moreover, further understanding of ACE2 characteristics and interactions with the virus and other possibly co-receptors in different species would provide useful insights into the genetic susceptibility to SARS-CoV-2 and address reasonable tracing of the viral zoonotic origin.

### ACE2 structure, tissue distribution, and multiple functions

**Fig. 1:**
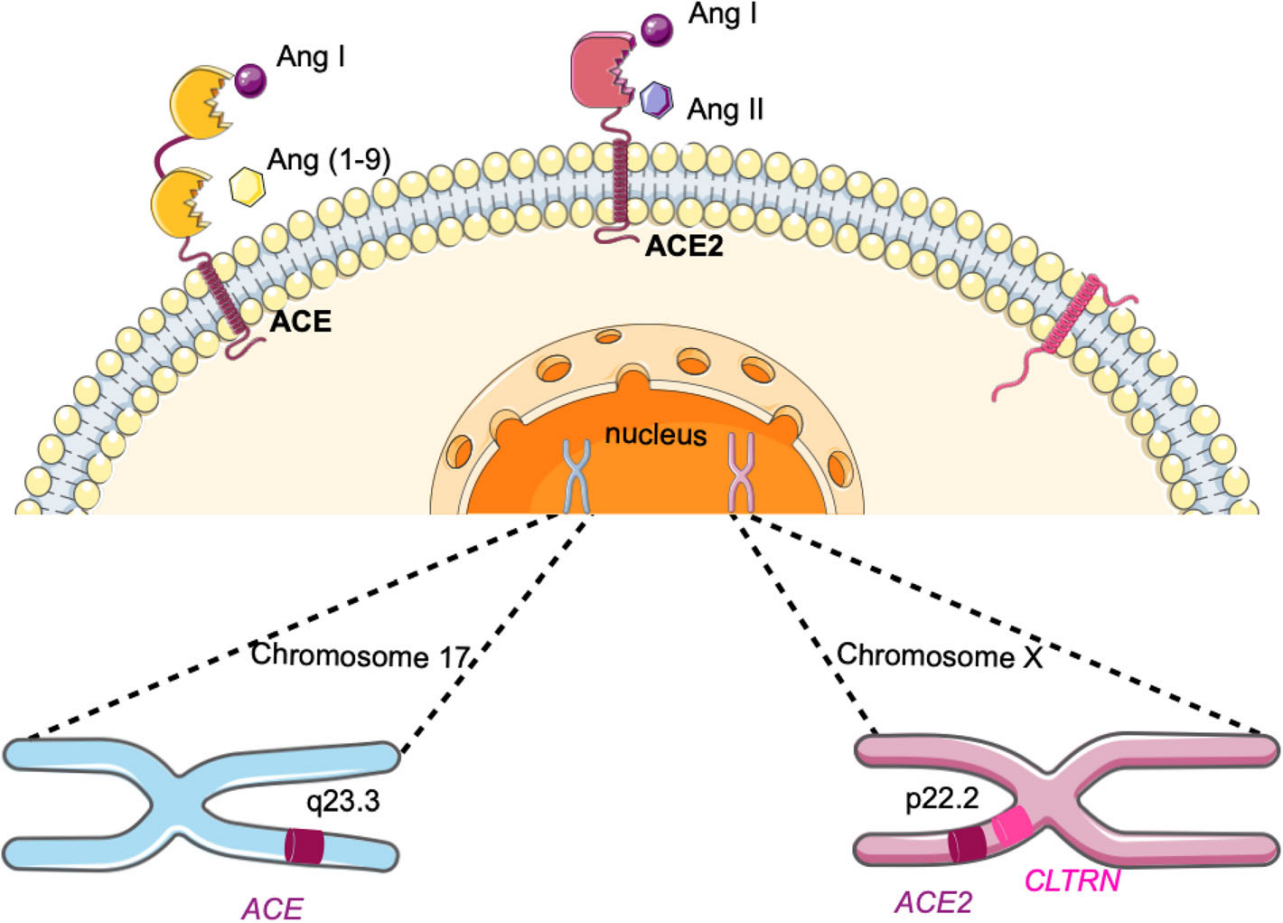
ACE, ACE2 and Collectrin chromosomal location and cellular localizations. The 3 type I transmembrane proteins ACE, ACE2, and collectrin located in the plasma membrane with activity differences. ACE acts as a dipeptidyl carboxypeptidase with 2 catalytic active sites for angiotensin I (Ang I) and angiotensin (1–9) (Ang (1–9)), whereas ACE2 acts as a monocarboxypeptidase possessing one active site that cleaves Ang I and angiotensin II (Ang II). However, collectrin lacks a catalytic activity in its extracellular domain. ACE2 and collectrin gene (CLTRN) are both mapped to chromosome Xp22.2 where ACE is located on chromosome 17q23.3

*ACE2* gene*,* mapped to chromosome Xp22 and 40 kb in size, constitutes 22 introns and 18 exons with a remarkable resemblance to *ACE*’s first 17 exons [4]. *ACE2* gene encodes a 120-KDa typical zinc-metalloproteinase type 1 transmembrane protein composed of 805 amino acids. ACE2 protein possesses a unique N-terminal catalytic domain on the extracellular surface and a C-terminal domain serving as a membrane anchor. Despite the considerable similarity between them, ACE and ACE2 do not function similarly and significant differences in their substrate specificities have been observed (Fig. 1). Structural protein studies have shown that both proteins exhibit a highly conserved catalytic domain and share a similar mechanism of action with a different substituted amino acid in the binding pocket [35, 36]. This substitution sterically hinders the access of the substrates to the ACE2 binding site leading to the elimination of ACE-like dipeptidase activity. Like carboxypeptidases, ACE removes C-terminal dipeptide to yield Ang II with injurious effects whereas ACE2 removes only one amino acid residue to produce Ang (1–7) and Ang (1–9) counterbalancing Ang II sequels [5]. Unexpectedly, the C-terminal cytoplasmic domain of ACE2 has high homology with the renal protein, collectrin, mapped on chromosome Xp22 as well, which acts as a molecular chaperone that binds to amino acid transporters, like the neutral amino acid transporter B^0^AT1, and regulates the trafficking of amino acids in the proximal tubules [37–39]. ACE2 was later shown to resemble a similar chaperone function. It heterodimerizes with B^0^AT1 in a tissue-specific manner and regulates membrane trafficking in the intestine where collectrin is absent [40–42].

**Fig. 2:**
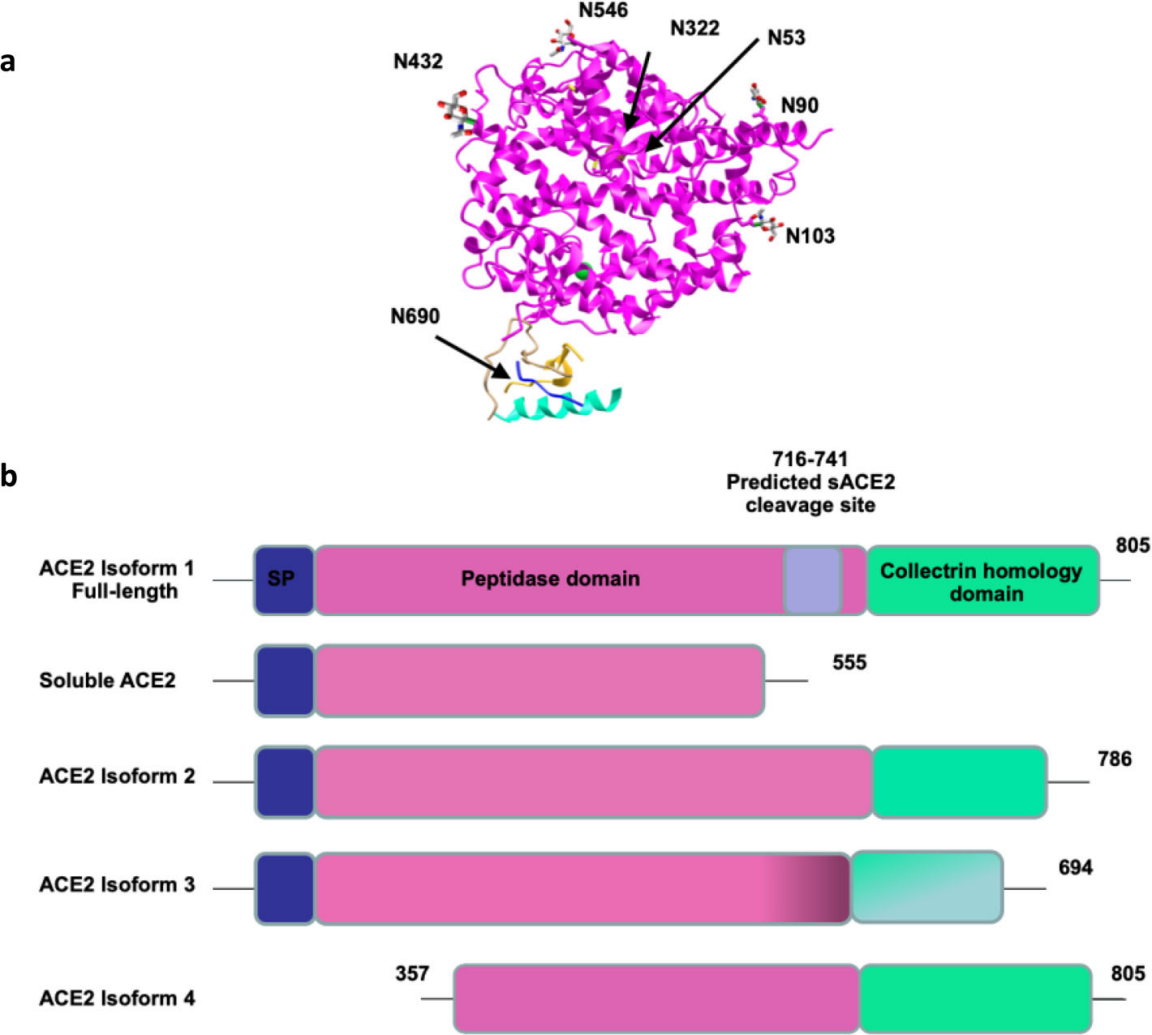
ACE2 3D-structure and isoforms. **a** 3D structure of ACE2 adopted from Towler et al. [36] and drawn in iCn3D using PBD ID: 1R4L. The structure was modified based on the newly identified N-glycosylation sites labeled in black. **b** ACE2 exists in 4 different isoforms along with a soluble form cleaved at residues 716–741 and constitutes 555 amino acids lacking the transmembrane collectrin homology domain. ACE2 isoform 2 shows a truncation of the full-length ACE2 with 100% homology in the 786 residues. ACE2 isoform 3 displays deletions in the transmembrane and collectrin homology domains leading to 95% similarity with ACE2 represented by gradient color in the corresponding domains. The fourth isoform with shorter N-terminus, lacking the carboxypeptidase activity and inability to bind to SARS-CoV-2. SP signal peptide

Moreover, two human ACE2 forms have been reported, the larger form corresponding to the full-length ACE2 with 18 exons and 805 amino acids and the shorter one corresponding to the soluble form of ACE2 (sACE2) which is 555 amino acids in size. sACE2 is obtained by shedding of the protein mainly through a disintegrin and metalloproteinase 17 (ADAM17) which is demonstrated to maintain its enzymatic activity and play a role in partially attenuating viral entry to the cells [43]. 3D structure analysis of ACE2 reveals a signal peptide sequence composed of 18 amino acids, an extracellular sequence (18-740) which contains the active carboxypeptidase domain, a transmembrane domain (741–761) and a cytoplasmic domain (762–805) which together the latter two form the collectrin homology domain (Fig. 2).

In addition, to date, 6 different transcript variants of human ACE2 are available in the GeneBank (transcript variant 1: NM_001371415.1, transcript variant 2: NM_021804.3, transcript variant 3: NM_001386259.1, transcript variant 4: NM_00138260.1, transcript variant 5: NM_001388425.1, and transcript variant 6: NM_001389402.1) corresponding to four ACE2 isoforms, other than sACE2, illustrated in Fig. 2b. Aside from the full-length isoform (805 amino acids) that is coded by transcript variants 1 and 2, three others have been reported. Transcript variant 3 encodes ACE2 isoform 2 that is a 786 amino acid protein. Based on blast sequence alignment, it is 100% identical with the full-length ACE2, where it is truncated at the cytoplasmic domain and lacks a collectrin homology domain, whereas the third ACE2 isoform (isoform 3) is generated by transcript variants 4 and 6 and is composed of 694 amino acids with 95% similarity with the full-length ACE2, where these transcripts lack some exons in the 3′UTR region and some deletions occur in the collectrin homology domain. It is worth noting that the role of these two isoforms in the SARS viral infections remains unidentified yet. However, a fourth isoform, known as dACE2 or MIRb-ACE2 has been identified, it lacks the carboxypeptidase activity and cannot bind to SARS-CoV-2. MIRb-ACE2 is a tissue-specific isoform and unstable truncated product. It has a shorter N-terminus with different 5′UTR and 5′ coding region compared to full-length ACE2 [44, 45].

ACE2 belongs to a family of transmembrane proteins that has wide tissue distribution. The observed difference in the cytoplasmic C-terminal sequence between ACE and ACE2 could explain the preferential localization of ACE2 on the apical membrane of polarized cells whilst ACE being localized on both the apical and basolateral membrane of epithelial cells [46]. Initial analysis has indicated that ACE2 expression is mainly in the rodent’s heart [3] and more precisely in cardiomyocytes and fibroblasts [47, 48]. However, in 2002, a detailed transcriptional profiling of ACE2 using real-time PCR has shown that transcribed ACE2 is expressed in 72 human tissues with higher expression in cardiovascular and renal systems [49]. Recently, bioinformatic analysis of publicly available data from human samples has generated extensive information about ACE2 distribution in tissues and cell types. Hikmet et al. have reported the expression pattern of ACE2 in more than 150 cell types, where the highest expression was observed mainly in the enterocytes, cardiomyocytes, and renal tubules and expression in the lungs was only limited to few subsets of cells [50]. Analysis of single-cell RNA-seq data has shown that ACE2 mRNA transcript is mostly detected in the alveolar type 2 cells of the lungs [51, 52]. The ACE2 expression profile was rarely investigated in COVID-19 patients, except for a study that displays an increased expression of mRNA in the nasopharyngeal and oropharyngeal swabs, with no data about its protein expression [34]. Furthermore, protein expression of ACE2 was initially identified in the heart, kidney, and testis [3–5], where immunolocalization studies have later demonstrated that it also exists on the surface of cells that are in contact with the cellular environment like the lung’s alveolar epithelial cells and the small intestine’s enterocytes [53]. ACE2 expression profiles were also affected in response to diseases. ACE2 protein expression analyzed by immunohistochemistry in bronchial and alveolar samples displayed a significant increase in type 2 diabetic subjects compared to controls, unlike its mRNA expression that was detected indifferent analyzed by RT-PCR [54]. In addition, ACE2 was also localized in the brain [55], islets of Langerhans of the pancreatic tissue [56] and bone [57] with no significant expression in the lymphatic system and lymphoid organs [53, 58].

### ACE2 polymorphic footprint in health and disease

In order to find an association with diseases, genetic variable signature has been extensively studied in different populations through single-nucleotide polymorphisms (SNPs). There are different factors, like age and ethnicity that affect the occurrence of SNPs and might consequently contribute to significant phenotypic changes and diseases’ outcomes. In this setting, ACE2 polymorphism and its association with hypertension were reported in different populations including the Chinese population with three major ACE2 variants (rs4830542, rs4240157, and rs4646155) [59], the Canadian population with another three different variants (rs233575, rs2074192, and rs2158083) [60], the Brazilian population with ACE2 G8790A mutation in combination with ACE I/D [61], and the Indian population with the ACE2 rs2106809 mutant [62]. It is not fully elucidated to date whether all these hypertension-related mutations affect the susceptibility/severity during SARS-CoV-2 infection. However, ACE2 rs2285666 was significantly correlated to lower infection rate in the Indian population [63] and ACE2 rs2074192 was significantly associated with severe COVID-19 outcomes in obese male smokers of age greater than 50 [64].

In an attempt to discover the association between COVID-19 and ACE2 polymorphic variations, being its major host cellular receptor, Cao and colleagues have investigated 1700 ACE2 coding variants collected from the China Metabolic Analytics Project and 1000 Genome Project databases. Their results demonstrated that no natural resistance mutations for SARS-CoV-2 S binding protein were detected in the studied populations. In addition, 32 variants were identified to potentially affect the amino acid sequence of ACE2 with 7 major variations unevenly distributed in different populations (Lys26Arg, Ile468Val, Ala627Val, Asn638Ser, Ser692Pro, Asn720Asp, and Leu731Ile/Leu731Phe) [65]. Notably, in another large population study, authors have identified natural ACE2 variants that might affect host susceptibility to SARS-CoV-2. Interestingly, they have also demonstrated that some variants potentially enhance susceptibility while others showed reduced binding to SARS-CoV-2 [66]. Among the ones that enhanced ACE2 affinity for the S protein is the Lys26Arg missense mutation. Lys26Arg along with another mutation, the Asn720Asp, were characterized by Al-Mulla and colleagues, in a preprint, as the most frequent missense variants of ACE2 in different global datasets [67]. Conversely, these results do not apply to an Italian COVID-19-positive population, where Novelli and colleagues have shown that no significant association is present between ACE2 and SARS-CoV-2 severity, speculating that susceptibility-related variants might be located in the non-coding region and contributing to the regulation of ACE2 activity [68]. Major ACE2 missense mutants involved in hypertension and COVID-19 are listed in Table 1. Of interest, none of these mutations has been clinically validated, where Human Gene Mutation Database (HGMD Professional 2020.3) displays only two reported ACE2 missense/nonsense mutations that are phenotypically related to autism spectrum disorder [69] and west syndrome [70].

**Table 1:** ACE2 variants’ association in different diseases

ACE2 variant	Clinical significance	Related diseases	Reference
rs2106809	-	Hypertension Metabolic diseases Diabetes mellitus type 2	[62, 71, 72]
rs2285666	-	Hypertension Diabetes mellitus type 2 COVID-19	[63, 73, 74]
rs2074192	-	Hypertension Diabetes mellitus type 2 Metabolic diseases COVID-19	[64, 71, 75, 76]
rs879922	-	Hypertension Diabetes mellitus type 2 Dyslipidemia Stroke	[62, 74, 75, 77]
rs1978124	-	Hypertension Diabetes mellitus type 2 Cardiovascular diseases	[74, 75]
rs4646155	-	Hypertension Dyslipidemia Cardiovascular diseases	[59, 74, 75]
rs4646156	-	Hypertension Diabetes mellitus type 2	[74, 75]
rs4240157	-	Hypertension Diabetes mellitus type 2 Cardiovascular diseases	[59, 74, 75]
rs233575	-	Hypertension Diabetes mellitus type 2 Cardiovascular diseases	[59, 74, 75]
rs6632677	-	Cardiovascular diseases	[78]
rs4646176	-	Hypertension	[71, 73]
rs4830542	-	Hypertension Dyslipidemia	[59, 74]
rs233575	-	Diabetes mellitus type 2 Dyslipidemia	[74, 75]
rs4646116	Likely-benign	COVID-19	[65, 67]
rs191860450	-	COVID-19	[65, 79]
rs784163894	-	COVID-19	[65]
rs183135788	-	COVID-19	[65]
rs149039346	-	COVID-19	[65, 80]
rs41303171	-	COVID-19	[65, 67]
rs147311723	-	COVID-19	[65, 80]

### Epigenetic variation of ACE2

The localization of the *ACE2* gene on the X chromosome raises the question of balance in the expression profiles between genders. Various studies have shown significant differences between males and females in ACE2 expression [81–84]. These findings were explained by identifying ACE2 as an escapee gene that undergoes incomplete X inactivation and shows a heterogenous sex-bias profile that is often shared across tissues [85]. X chromosome inactivation was shown to be greatly affected by epigenetic variations like the DNA-methylation that also affects the ACE2 expression profile [86].

Modifications in the DNA and chromatin structures are marked as epigenetic variations that were shown to play an important role in several human diseases like cardiovascular ones [87, 88]. Interestingly, recent studies have suggested that ACE2 production rate is controlled by its epigenetic modifications, where methylation of ACE2 gene near the transcription start site is found to be associated with the age- and gender-dependent variations of ACE2 with the lowest rate of methylation in the lungs and the highest in neurons where ACE2 protein is not detected [89]. Along these findings, ACE2 profiles were also shown to display a significant correlation with histone modification-related genes in the human lungs [90]. Additionally, the results of another study have previously demonstrated that ACE2 promoter was hypermethylated in hypertensive patients with a significant difference between males and females [91], where COVID-19 patients as well have displayed differential methylation pattern in ACE2 of blood samples [92] which further requires testing in the respiratory samples.

Aside from the pre-transcriptional regulation, the ACE2 mRNA level displays an epigenetic signature through the putative short non-coding micro-RNA regulation network. Several studies have reported new miRNAs that are involved with ACE2 expression either via hampering its translation or through degrading its corresponding protein. A recent bioinformatic study has identified 1954 miRNAs involved in the ACE2 regulating network [93]. miRNAs were either directly regulating ACE2 like miR-421, miR-125b, and miR-483-3p via having a putative site in its 3′UTR region [88, 94–96] or indirectly affecting ACE2 expressions like miR-181a and miR-4262 through affecting RAS components and target proteins (apoptotic Bcl2), respectively [97, 98]. A list of the microRNAs that target ACE2 is displayed in Table 2.

**Table 2:** microRNAs targeting angiotensin-converting enzyme (ACE2)

miRNA	Effect on ACE2	Reference
miR-18a	Inhibiting miR18a partially blocked ACE2 beneficial effect in hypoxia/reoxygenation endothelial cell model.	[99]
miR-21	Ang II-induced miR-21 mediates the inhibition of ACE2 antifibrotic effect in lung fibroblasts.	[100]
miR-29	Increased ACE2 expression in cardiac myocytes/fibroblasts during hypertrophic cardiomyopathy	[101]
miR-98 and miR-223	Downregulation of miR-98 and miR-223 leads to reduced expression of ACE2 in bronchial stem cells during SARS infection.	[102]
miR-125b	High glucose-induced upregulation of miR-125b in renal tubular epithelial cells leads to reduced ACE2 expression during diabetic nephropathy.	[95]
miR-483-3p	AT1R-regulated expression of miR-483-3p regulates the expression of major RAS components, including ACE2.	[94]
miR-421	Enhanced miR-421 expression in uremic patients downregulates ACE2 expression in the leukocytes during chronic kidney disease. Increased expression of miR-421 in myofibroblasts also downregulates ACE2 expression in thrombosis.	[96, 103]
miR-143	Downregulated miR-143 induced by aerobic exercise training was accompanied by increased ACE2 expression in hypertensive rats.	[104]
miR-4262	During acute lung injury, increased ACE2 expression suppresses miR-4262 leading to apoptotic Bcl2 upregulation and consequently inhibiting apoptosis.	[98]
miR-9-5p and miR-218-5p	Bioinformatic prediction algorithms identify miR-9-5p and miR-218-5p as regulators of SARS-CoV-2 through binding to 3′UTR region of ACE2.	[105]

In addition, other factors like smoking and sex hormone can also induce epigenetic modifications in the genome. Several studies conducted on the sex hormone have shown a correlation with ACE2 expression level which could also contribute to the sex-biased susceptibility of COVID-19. The female sex steroid 17β-estradiol (E_2_) was shown to regulate ACE2 expression, where it induces downregulation in the kidney and the differentiated airway epithelial cells [83, 106]. Conversely, in the atrial heart tissue, ACE2 expression was upregulated through the E2-estrogen receptor alpha-activated pathway [107]. In addition, another study on the male hormone, testosterone, was reported to upregulate ACE2 expression [108]. Furthermore, cigarette smoking was demonstrated to enhance ACE2 expression which also presented a risk factor for the progression of COVID-19 with more severe complications [109, 110].

### Subcellular localization and intracellular trafficking of ACE2

Among the 10% proteins that are destined to the plasma membrane [111], nascently biosynthesized ACE2 traffics to its intended location in the cell via a highly regulated machinery that includes different subcellular compartments. Translated proteins are inserted into the endoplasmic reticulum (ER) via their signal peptides, where they undergo folding into their correct conformations. Post-translational modifications of the proteins, such as attaching carbohydrate moieties, a process named glycosylation which is a common modification of secretory pathway targeted proteins, is initiated in the ER. In order to exit the ER, proteins need to be fully folded and assembled (in case of multi-subunit complexes) before they are allowed to exit the ER. Misfolded proteins and orphaned subunits of protein complexes are retained in the ER and transported to the cytosol where they are degraded by the proteasome through ERAD (endoplasmic reticulum-associated protein degradation) degradation process [112, 113]. At the ER exist sites, folded proteins are transported from the ER by transport vesicles into the Golgi apparatus through the cis Golgi network. Proteins are further processed in the Golgi including carbohydrate remodeling, sulfation, proteolysis, and phosphorylation. Processed proteins are then sorted and exit the Golgi through the trans-Golgi network via the secretory pathway in which they are secreted or targeted to subcellular compartments such as the plasma membrane [114].

**Fig. 3:**
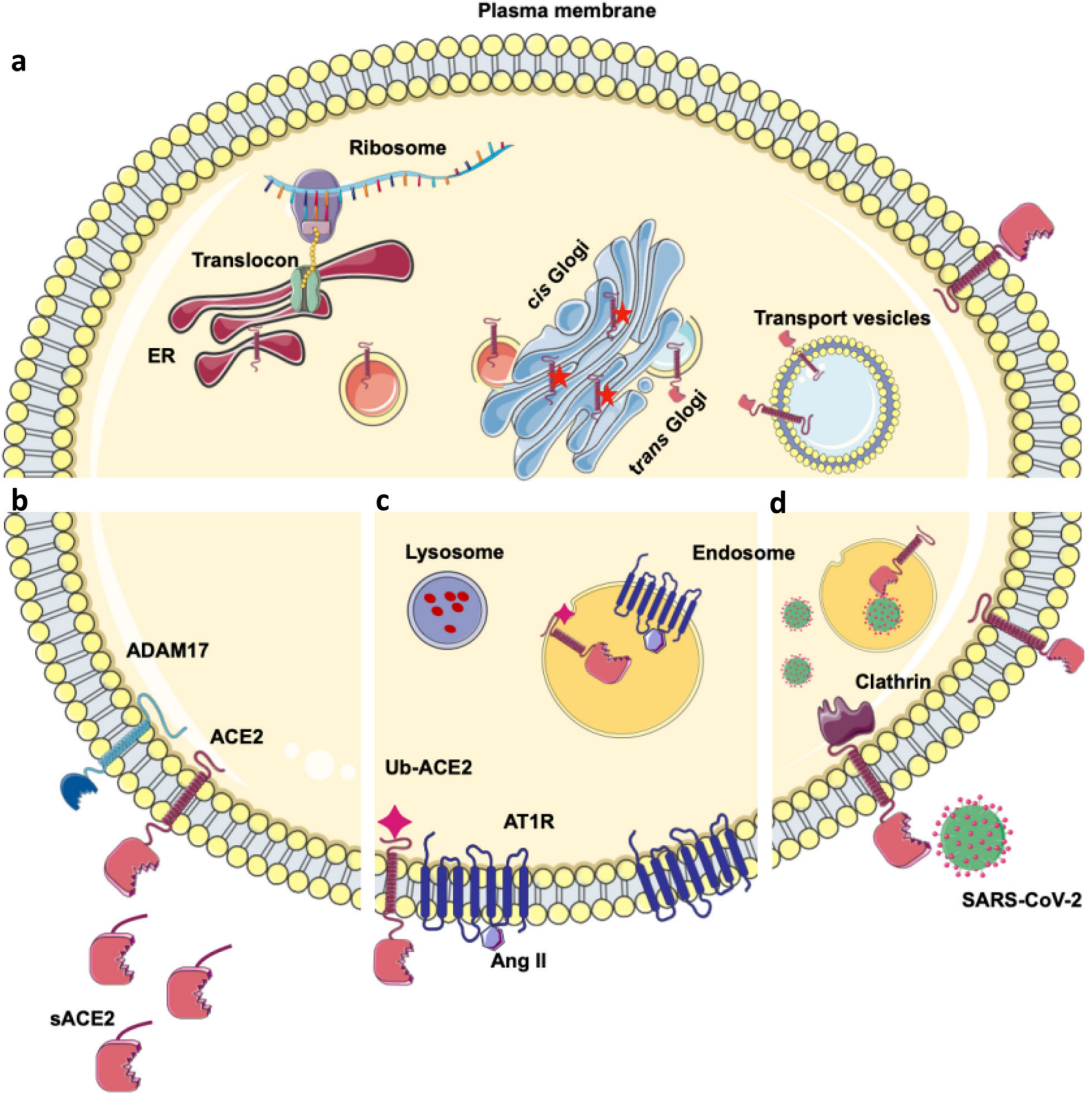
ACE2 synthesis, trafficking, proteolysis, and internalization. **a** Synthesis of ACE2 protein and its translocation to the endoplasmic reticulum (ER) through Golgi apparatus towards the plasma membrane via transport vesicles. Red stars correspond to post-translational modifications. **b** Truncation of ACE2 by ADAM17 and the release of soluble ACE2 (sACE2) into the extracellular environment. **c** Internalization of ACE2 in response to increased angiotensin II (Ang II) via ubiquitylation of ACE2 and interaction with angiotensin type I receptor (AT1R). The latter complex is endocytosed where ACE2 is degraded by the lysosome and AT1R is recycled and transported back to the membrane. **d** ACE2 internalization in response to SARS-CoV-2 binding via clathrin-mediated endocytosis. ACE2 is recycled and transported to the membrane and SARS-CoV-2 is replicated inside the host cell

Generally, proteins composed of more than 100 amino acids, including ACE2, undergo co-translational translocation into the ER while being translated. Unlike the small proteins that cross the ER membrane, newly synthesized ACE2 is targeted to the translocon (ER membrane channel) via its 17 amino acid N-terminal signal sequence. As translation proceeds, ACE2 binds to the channel and passes to the ER membrane where the ribosome is dissociated [115]. Once ACE2 is well folded and N-glycosylated in the ER, it exits through transport vesicles and enters the Golgi apparatus through its cis face. In the Golgi, ACE2 undergoes further modifications and packaging and is then transported to the plasma membrane by vesicular transport (Fig. 3a) [116]. As mentioned previously, the intracellular localization of a protein to the plasma membrane is a highly regulated machinery that requires interaction with accessory proteins. Unfortunately, ACE2 accessory proteins are not yet investigated, except for the actin-bundling protein fascin-1 that has displayed differential interaction with ACE2 in the HEK293T cell model in an Ang II-dependent manner [117]. Characterizing these interactors and deciphering their role during intracellular trafficking might represent a promising therapeutic tool to decrease ACE2 availability at the cellular membrane.

To date, several glycosylation sites have been identified on ACE2. In fact, seven asparagine residues were detected to undergo N-glycosylation in human ACE2 (N53, N90, N103, N322, N432, N546, and N690) (Fig. 2a) [118]. The same study has suggested that glycosylation of ACE2 had no effect on its binding affinity with the S protein of SARS-CoV-2. However, these data contradict other studies that have reported N90 and N322 glycosylation to be interfering with the binding and contributing significantly to the infectivity of the virus [119, 120]. Proper N-glycosylation is expected to be important for the efficient trafficking of the receptor to the plasma membrane. However, a study by Zhao and colleagues showed that inhibiting ER-resident glucosidases, responsible for trimming sugars prior to protein folding, altered the glycan structure of ACE2. This alteration did not affect neither ACE2 cellular expression nor its binding to SARS-CoV, but it showed impaired ability in the viral-induced membrane fusion [121]. Similarly, in the context of glycosylation, a study by Vincent and colleagues have demonstrated that effective doses’ treatments of chloroquine and NH_4_CL, not only affected the viral proteins, but also induced impaired terminal glycosylation of ACE2 and increased intracellular mobility in the ER and the Golgi. However, these modifications displayed no effect on ACE2 localization to the cellular surface [122]. Moreover, modifications other than glycosylation like methylation have also been identified on human ACE2 at different sites, unlike phosphorylation and acetylation post translational modifications that were not detected [118].

Type I transmembrane proteins are subjected to shedding, also known as proteolytic cleavage, where the protein’s ectodomain is cleaved by a protease and is released extracellularly in order to control the protein’s expression and function [123]. ACE2 is catalytically cleaved by ADAM17, a metalloprotease family member, near the transmembrane domain, between the residues 716 and 741, leading to an enzymatically active soluble ACE2 (Fig. 3b) [43, 124]. Interestingly, a mutation at the 584 residues has inhibited the shedding activity but did not affect the trafficking of the mutant ACE2 to the cell surface, noting that several mutations at different residues have displayed no effect (580, 581, 582, 583, and 604) neither on ACE2 shedding nor surface targeting [43]. The shedding event of ACE2 is regulated by different stimuli. Increased soluble ACE2 levels have been reported in cardiovascular diseases contributing to higher blood pressure [125]. In addition, spike protein binding to the ACE2 receptor induces its cleavage; however, it does not augment the viral infectivity [126]. TMPRSS2, type II transmembrane serine protease, was demonstrated to have a competitive cleavage activity that removes a C-terminal fragment of ACE2 and contributes to further virulence during SARS-CoV infections [126–128].

Furthermore, ACE2 was shown to display an internalization pattern under different stimulations. During hypertension, decreased ACE2 protein expression level contributed to an internalization compensatory mechanism in response to increased Ang II, mediated through the angiotensin II type I receptor (AT1R) [129]. ACE2 displayed enhanced ubiquitination and interacted with AT1R, where the latter is recycled and transported back to the membrane via endosomes, whereas ACE2 was degraded in the lysosome (Fig. 3c). In addition, ACE2 was also internalized during SAR-CoV-1 and SARS-CoV-2 infections via a clathrin-mediated endocytosis [130, 131] in which it was suggested that ACE2 is recycled back to the cell surface and the virus is further replicated in the cell (Fig. 3d) [130].

### ACE2 as a therapeutic target

Given the protective effect that it displays, ACE2 represents a potent therapeutic target to prevent and treat several cardiovascular diseases such as hypertension. Strategies that aim to enhance the protective role of ACE2 like ACE inhibitors and angiotensin-receptor blockers (ARB) have shown effectiveness in treating high blood pressure and some other cardiovascular issues. In this context, treatments were based on activating ACE2. However, researchers have developed a new strategy based on exogenous administration of ACE2 in which recombinant human ACE2 (rhACE2) was used and demonstrated encouraging cardioprotective, anti-fibrotic effects, and protection against lung injury [15, 132, 133].

Paradoxically, ACE2 acts as a double-edged sword where this protective effect is abolished in the presence of SARS viral infections. Conversely, therapeutic strategies could aim to decrease ACE2 expression or alter its binding affinity to SARS S protein and consequently reduce the viral entry to host cells. Pharmacologic RAS inhibition through ACE inhibitors or ARBs was hypothesized to upregulate ACE2 in diabetic and hypertensive patients which will subsequently amplify the viral infection [134]. However, the concerns regarding the potential harmful effect of ACE inhibitors and ARBs were not confirmed to be true. A study by Peng and colleagues has shown that the use of ACEI/ARBs does not affect the mortality rate in cardiovascular patients infected with COVID-19 [135]. Furthermore, another study has demonstrated that RAS inhibition significantly contributes to lower virulence [136]. Interestingly, the administration of rhACE2 to SARS-CoV-2 patients could display a positive approach due to its hypothetical dual function. Increasing ACE2 availability could contribute to slowing down viral entry through its competitive binding to the viral S protein and could protect the lung against the subsequent injury through its classical protective role [137]. Monteil and colleagues have previously demonstrated that soluble rhACE2 was able to alter the early infection stages of SARS-CoV-2 in engineered human kidney organoids [138]. Noting that it was not tested in any animal model [139], the use of recombinant human ACE2 was remarkably tolerated at different doses in healthy human subjects and patients with acute respiratory distress syndrome [16, 17]. To date, rhACE2 (APN01) is being assessed as a treatment for patients with SARS-CoV-2 infection. Currently, the pilot clinical study has 200 participants and is in phase 2 clinical trial [18]. In this context, genetically modified mouse models have been proposed to enhance preclinical studies in COVID-19 research [140].

Additionally, in a new nanotechnology approach, it was suggested that ACE2 nanoparticles applied to the protective personal equipment (masks, gloves, and clothes) could present an effective strategy in tackling the virus and preventing its entry to the host cells [141]. Moreover, none of these strategies has been approved yet and further studies are still required.

## Discussion

Given the multifunction, complexity, and dynamic nature of ACE2, the present understanding of its structure and function represents the beginning of guidance into therapeutic solutions. The presence of conflicting data highlights the importance of systems biology studies of the viral infections that include different variables to provide a holistic perspective of how our system interacts and responds to SARS-CoV-2 infection, noting its wide distribution in human tissues [49, 50]. Reductionist studies of ACE2 have led to a massive accumulation of data; however, unfortunately, no effective medication for COVID-19 is available to date. After its synthesis, ACE2 is subjected to different interactions and regulations that might be occurring simultaneously and not separately, affecting its cellular trafficking, localization, and expression. These interactions might differ between individuals based on their genetic signature and consequently may lead to variable virulence and infectivity.

The presence of genetic variations in the host cellular receptor could greatly contribute to the observed variable susceptibility of SARS-CoV-2 infection. Their occurrence in the promoter region of the ACE2 gene could consequently lead to decreasing its cellular expression. Moreover, the presence of these variants in the coding region would probably lead to altering its amino acid sequence that might modify its structure and alter its plasma membrane targeting and as a result reduce the interaction with SARS-CoV-2 S protein. The latter has been also shown in a study by Guo et al. where different missense mutations were shown to affect ACE2 secondary structure and weaken its activity [79]. A recently published study that investigates the impact of ACE2 mutants on COVID-19 susceptibility shows that ACE2 SNPs could greatly influence its folding, its expression, and its interacting miRNA and consequently affecting the viral susceptibility [80]. Compared to ACE2, a point mutation on the 1069 residue of ACE, located in the C-terminal domain, is reported to be responsible for autosomal renal tubular dysgenesis (RTD) disease. This mutation has led to retaining ACE in the ER and increased its degradation leading consequently to its decreased cell surface localization [142]. Besides, ACE was reported to interact with immunoglobulin-binding protein (BiP) chaperone that resides in the ER, its overexpression leads to the retention of ACE in the ER and decreases its cell surface expression which suggests transient interaction with BiP for optimal transport. This study has demonstrated that BiP affects exclusively the transport of ACE rather than its synthesis [143]. Moreover, the use of pharmacological chaperones and proteasome inhibitors prevented intracellular degradation and rescued mutant ACE to the plasma membrane [144]. Additionally, in the context of RTD, several mutations at different residues were evaluated. Missense (at 594 and 828 residues) and truncated mutants (at 1136 and 1145 residues) were also retained in ER and displayed no plasma membrane expression, where another mutant at 1180 has displayed partial ER retention and delayed cell surface expression compared to wild type-ACE [145]. Whether the different identified mutations and isoforms of ACE2 could modify its trafficking and lead to cellular retention is not tested yet. Recently, Gurumurthy et al. have proposed different genetically engineered mouse models that can be used to engineer the different ACE2 identified mutations in vivo and assess their effects [140]. In addition, a combination of these mutants could also occur together leading to a further decreased ACE2 membrane expression. In a deep mutagenesis study involving the soluble ACE2, combining different engineered single mutations together showed higher binding to the spike protein of SARS-CoV-2 [146]. Understanding the trafficking and secretory pathways of classical and mutated ACE2 shall provide potential trafficking modulators that can be targeted to improve clinical outcomes.

## Conclusions

In summary, “in every angel a demon hides and in every demon an angel strides.” The angelic protective role of ACE2 is interchanged with the emergence of SARS viral infections and the evil ACE2 as a host cellular receptor can potently be reciprocated and act as a therapeutic target to treat COVID-19 patients. Through this review, we highlight the importance of further mutational screening, trafficking assessment, and systems biology studies of ACE2 and their role in the generation of a unique individual fingerprint that might explain why some people are more susceptible to SARS-CoV-2 and others are not. Consequently, this could help us understand the downstream mechanism of ACE2-mediated infection and possibly characterize novel therapeutic strategies for tackling COVID-19.

## Data Availability

Data sharing is not applicable to this article as no datasets were generated or analyzed during the current study.

## Abbreviations

ACE: Angiotensin-converting enzyme
ACE2: Angiotensin-converting enzyme 2
ACEI: Angiotensin-converting enzyme Inhibitor
ADAM17: A disintegrin and metalloproteinase 17
Ang II: Angiotensin II
Ang(1–7): Angiotensin (1–7)
Ang(1–9): Angiotensin (1–9)
ARB: Angiotensin receptor blocker
AT1R: Angiotensin II type I receptor
B0AT1: Neutral amino acid transporter
BiP: Binding immunoglobulin protein
CLTRN: Collectrin
COVID-19: Coronavirus disease-2019
ER: Endoplasmic reticulum
I/D: Insertion/deletion
RAS: Renin-angiotensin system
rhACE2: Recombinant human angiotensin-converting enzyme 2
RTD: Renal tubular dysgenesis
sACE2: Soluble angiotensin-converting enzyme 2
SARS-CoV-2: Severe acute respiratory syndrome - coronavirus 2
SNP: Single nucleotide polymorphism
SP: Signal peptide
TMPRSS2: Type II transmembrane serine protease
